# Prolyl Endopeptidase Gene Disruption Improves Gut Dysbiosis and Non-alcoholic Fatty Liver Disease in Mice Induced by a High-Fat Diet

**DOI:** 10.3389/fcell.2021.628143

**Published:** 2021-05-20

**Authors:** Daixi Jiang, Jianbin Zhang, Shuangzhe Lin, Yuqin Wang, Yuanwen Chen, Jiangao Fan

**Affiliations:** ^1^Department of Gastroenterology, Xinhua Hospital Affiliated to Shanghai Jiao Tong University School of Medicine, Shanghai, China; ^2^State Key Laboratory for Diagnosis and Treatment of Infectious Diseases, National Clinical Research Center for Infectious Diseases, Collaborative Innovation Center for Diagnosis and Treatment of Infectious Diseases, The First Affiliated Hospital, Zhejiang University School of Medicine, Hangzhou, China

**Keywords:** liver, gut microbiota, non-alcoholic fatty liver disease (NAFLD), gene knockout, prolyl endopeptidase

## Abstract

The gut-liver axis is increasingly recognized as being involved in the pathogenesis and progression of non-alcoholic fatty liver disease (NAFLD). Prolyl endopeptidase (PREP) plays a role in gut metabolic homeostasis and neurodegenerative diseases. We investigated the role of PREP disruption in the crosstalk between gut flora and hepatic steatosis or inflammation in mice with NAFLD. Wild-type mice (WT) and PREP gene knocked mice (PREP^gt^) were fed a low-fat diet (LFD) or high-fat diet (HFD) for 16 or 24 weeks. Murine gut microbiota profiles were generated at 16 or 24 weeks. Liver lipogenesis-associated molecules and their upstream mediators, AMP-activated protein kinase (AMPK) and sirtuin1 (SIRT1), were detected using RT-PCR or western blot in all mice. Inflammatory triggers and mediators from the gut or infiltrated inflammatory cells and signal mediators, such as p-ERK and p-p65, were determined. We found that PREP disruption modulated microbiota composition and altered the abundance of several beneficial bacteria such as the butyrate-producing bacteria in mice fed a HFD for 16 or 24 weeks. The level of butyrate in HFD-PREP^gt^ mice significantly increased compared with that of the HFD-WT mice at 16 weeks. Interestingly, PREP disruption inhibited p-ERK and p-p65 and reduced the levels of proinflammatory cytokines in response to endotoxin and proline-glycine-proline, which guided macrophage/neutrophil infiltration in mice fed a HFD for 24 weeks. However, at 16 weeks, PREP disruption, other than regulating hepatic inflammation, displayed improved liver lipogenesis and AMPK/SIRT1 signaling. PREP disruption may target multiple hepatic mechanisms related to the liver, gut, and microbiota, displaying a dynamic role in hepatic steatosis and inflammation during NAFLD. PREP might serve as a therapeutic target for NAFLD.

## Introduction

Non-alcoholic fatty liver disease (NAFLD) is a growing global health concern that affects around one-fourth of the general population worldwide (Younossi et al., 2016). The spectrum of NAFLD consists of non-alcoholic fatty liver (NAFL), the more advanced stage non-alcoholic steatohepatitis (NASH), NASH-related cirrhosis, and hepatocellular carcinoma (Saltzman et al., 2018; Aron-Wisnewsky et al., 2020). NAFLD pathogenesis is highly complex and involves numerous pathways, including insulin resistance, inflammation, lipotoxicity, increased *de novo* lipogenesis, oxidative stress, and gut dysbiosis (Tilg and Moschen, 2010; Suzuki and Diehl, 2017; Lonardo et al., 2018). Several factors, likely acting in parallel, contribute to NAFLD development and progression. These factors need to be better understood since no effective drug regimen that completely reverses the disease is currently available (Tilg and Moschen, 2010; Younossi et al., 2018).

A new model called “multiple organs-multiple hits” was proposed to explain NASH progression mechanisms (Schuster et al., 2018; Yan et al., 2020). A growing body of experimental and clinical evidence suggests that gut microbiota may be implicated in NAFLD pathogenesis (Abu-Shanab and Quigley, 2010; Safari and Gerard, 2019). Recently, studies found that certain plant extracts with prolyl endopeptidase (PREP) inhibitory function exert both intestinal flora and anti-NAFLD/NASH effects (Chen et al., 2014; Babkova et al., 2017; Wang et al., 2017). Consumption of chlorogenic acid (often through coffee) benefits intestinal functions and regulates the abundance of certain bacteria in the cecum (Chen et al., 2019). Berberine, commonly used for treating diarrhea in China (Kong et al., 2004; Yan et al., 2020), could induce gut microbiota-derived bioactive metabolite production, including butyrate, ultimately improving energy metabolism (Wang et al., 2017). It is worth mentioning that these extracts are naturally occurring PREP inhibitors (Adolpho et al., 2013; Babkova et al., 2017). As mechanisms may vary via different pathways in NAFLD development, various PREP roles in different organs need to be identified for further therapeutic applications.

Plant extracts with prolyl endopeptidase belongs to a unique family of serine proteases that specifically hydrolyze prolyl-containing bioactive peptides at the C-termini of proline residues (Shan et al., 2005). PREP is mainly found in the brain (Myohanen et al., 2007); however, significant PREP activities and protein levels have been measured in peripheral tissues, such as the liver and colorectal tumors (Larrinaga et al., 2014). One study has reported a beneficial effect of PREP in the intestine. PREP induction translated gluten into gluten immunogenic peptides in the intestine, thus improving metabolic homeostasis in mice fed a high-fat diet (HFD) (Olivares et al., 2019). However, another study showed PREP detrimental effect when collagen was cleaved by matrix metalloproteinases and PREP into proline-glycine-proline (PGP), which guided neutrophilic infiltration in the intestine and induced a vicious cycle in neutrophilic inflammation in the context of inflammatory bowel disease (Koelink et al., 2014). Our previous work found that N-acetyl-seryl-aspartyl-lysyl-proline (AcSDKP), generated from thymosin β4 (*T*β4) through hydrolysis of meprin-α and PREP, exerts a therapeutic effect on inflammatory bowel disease (Shi et al., 2020). Our studies also indicated that PREP inhibition improves hepatocyte steatosis *in vitro* and *in vivo* (Zhou et al., 2016; Jiang et al., 2020). However, the interactions between PREP and the gut environment in HFD-induced NAFLD and their potential multi-organ mechanisms remain unknown.

Herein, we conducted *in vivo* experiments at different times and in different organs to explore the role of PREP disruption on HFD-induced steatohepatitis, focusing on its controversial role in gut flora and its relationship with HFD-induced hepatic steatosis and inflammatory responses, and to elucidate its possible mechanism of action.

## Materials and Methods

### Animal Model and Diets

Wild-type (WT) C57BL/6J and PREP-disrupted (PREP^gt^) mice were obtained from the Shanghai Model Organisms Center, Inc. The details of PREP knockout mice are provided in the Methods section of our previous study (Jiang et al., 2020). Mice were fed a standard chow diet or a HFD (fat 30 kcal%, carbohydrates 52 kcal%, protein 18 kcal%, and cholesterol 2%) for 16 or 24 weeks. All mice were housed under a 12:12 h light/dark cycle at 25 ± 2°C and were allowed free access to food and water. All animal experiments followed the National Research Council’s Guide for the Care and Use of Laboratory Animals and were approved by the Institutional Animal Care and Use Committee of SHRM (SHRM-IACUC-022).

### Gut Microbiota Analysis

Cecal content samples were snap-frozen and stored at −80°C. Bacterial DNA was isolated from the cecal contents using a DNeasy PowerSoil kit (Qiagen, Hilden, Germany) according to the manufacturer’s protocols. The quality and quantity of DNA were measured using a NanoDrop 2000 spectrophotometer (Thermo Fisher Scientific, Waltham, United States) and agarose gel electrophoresis, respectively. The V3–V4 regions of the bacterial 16S ribosomal RNA gene were amplified in a 25-μl reaction using PCR. The amplicons were purified using Agencourt AMPure XP beads (Beckman Coulter Co., United States). Purified amplicons were then applied to the Illumina MiSeq platform (Illumina Inc., San Diego, United States). After paired-end reads were preprocessed using Trimmomatic software (Bolger et al., 2014) to detect and cut off ambiguous bases, FLASH software was used to assemble paired-end reads (Reyon et al., 2012). All results were based on sequenced reads and operational taxonomic units (OTUs).

### Hematoxylin and Eosin and Immunohistochemistry Staining

Liver tissue and ileum were fixed in 4% paraformaldehyde at 4°C overnight, then embedded in paraffin wax or snap-frozen in liquid nitrogen and stored at −80°C. Paraffin sections were stained with hematoxylin-eosin (H&E) for pathological analysis. NALFD activity score (NAS) is calculated from the semi-quantitative evaluation of hepatic steatosis, lobular inflammation, and hepatocyte ballooning, as the previous review concluded (Aron-Wisnewsky et al., 2020). For immunohistochemistry, liver sections were incubated with antibodies against F4/80 (gb11027, Servicebio, China) and myeloperoxidase (MPO) (gb11224, Servicebio, China). The number of positive cells in the liver sections was normalized to the tissue area. Ileum sections were incubated with antibodies against zonula occludens 1 (ZO-1) (ab96587, Abcam, United Kingdom) and occludin (ab216327, Abcam, United Kingdom). Images were captured using an optical microscope (Olympus BX51, China).

### PREP Activity Fluorometric Assay

The PREP activity assay was performed as described previously (Zhou et al., 2016). Briefly, 60 mg liver tissues were homogenized in 500 μl assay buffer (10 mmol/L Tris–HCl buffer, pH 7.4) and then centrifuged for 20 min at 4°C. Thereafter, 465 μl Tris–HCl (pH 7.4) was added to 10 μl supernatant for 30 min at 37°C. Next, 25 μl of the substrate (4 mmol/L Suc-Gly-Pro-AMC, Bachem) was added. The reagents were mixed, and the reaction was incubated for 60 min at 37°C. After adding the stop solution (500 μl, 1 mol/L sodium acetate buffer, pH 4.2), the fluorescence intensity was read at Ex/Em = 360/460 nm. The final concentrations were normalized to protein content and reaction time.

### MMP9 Fluorometric Assays

Liver tissue samples were homogenized in assay buffer and centrifuged for 15 min at 10,000 × *g* 4°C, followed by their activation with APMA (1 mM; AnaSpec, United States) for 2 h at 37°C. The active MMP-9 was detected using SensoLyte 520 MMP-9 Assay Kit (fluorometric) using a 5-FAM/QXL^TM^520 fluorescence resonance energy transfer peptide (AS-71155, AnaSpec, United States), according to the manufacturer’s instructions. The reagents were mixed, and the fluorescence intensity was read at Ex/Em = 490/520 nm after adding the stop solution. The final concentrations were normalized to protein content.

### Immunoblots

Liver or ileum tissue was homogenized and lysed in ice-cold RIPA lysis buffer (Beyotime, Shanghai, China). Total protein concentrations were measured using the BCA Protein Assay Kit (Beyotime, Shanghai, China). For immunoblotting, the protein extracts were loaded onto SDS-polyacrylamide gels (SDS-PAGE) and separated. Then, the proteins were transferred onto polyvinylidene difluoride membranes and blocked with 5% skimmed milk. Next, the membranes were incubated with primary antibodies, followed by incubation with secondary antibodies and enhanced chemiluminescence. Antibodies against sirtuin1 (SIRT1, 9475T), phosphorylated-adenosine 5′-monophosphate-activated protein kinase (PAMPK, 2535T), AMPK (4150P), fatty acid synthase (FAS, 3180S), phosphorylated-p65 (3033P), p65 (8242P), phosphorylated-ERK1/2 (4370T), and ERK1/2 (4695T) were obtained from Cell Signaling Technology (Beverly, MA, United States). Antibodies against sterol regulatory element-binding transcription factor 1 (SREBP1 and GB11524) and GAPDH (GB11002) were obtained from Servicebio (Wuhan, China). Antibodies against MMP9 (ab38898) were obtained from Abcam. The bands were quantified using Image Lab Version 2.0.1 (Bio-Rad, Hercules, CA, United States). The western blots used for analysis are included in the Supplementary Files (Supplementary Figures 3, 4).

### Quantitative Reverse Transcription-Polymerase Chain Reaction (RT-PCR) Analysis

The tissue samples were homogenized using TRIzol reagent (Takara, Dalian, China) to extract total RNAs, which were reverse transcribed to cDNA using reverse transcriptase (Takara, Dalian, China). Thereafter, cDNA was used to perform real-time PCR using SYBR Premix Ex Taq (Tli RNase H Plus) (Takara, Dalian, China) using a ViiA7 real-time PCR system (Applied Biosystems, United States). Glyceraldehyde 3-phosphate dehydrogenase (*GAPDH*) was used as an internal control. Relative mRNA expression levels were determined using the 2^−ΔΔCt^ method. The gene-specific primers in this experiment are listed in Supplementary Table 1.

### Statistical Analysis

All data are expressed as the means ± SEM. Comparisons were performed using a one-way analysis of variance (ANOVA) in GraphPad Prism 6 (GraphPad Software Inc., San Diego, CA, United States). Tukey’s post-hoc comparisons were applied for comparisons between multiple experimental groups. Differences were considered significant at *P*-values < 0.05.

## Results

### Hepatic Steatosis and Liver Injury Were Ameliorated by PREP Disruption in the Liver of HFD-Fed Mice at Different Time Points

**FIGURE 1:**
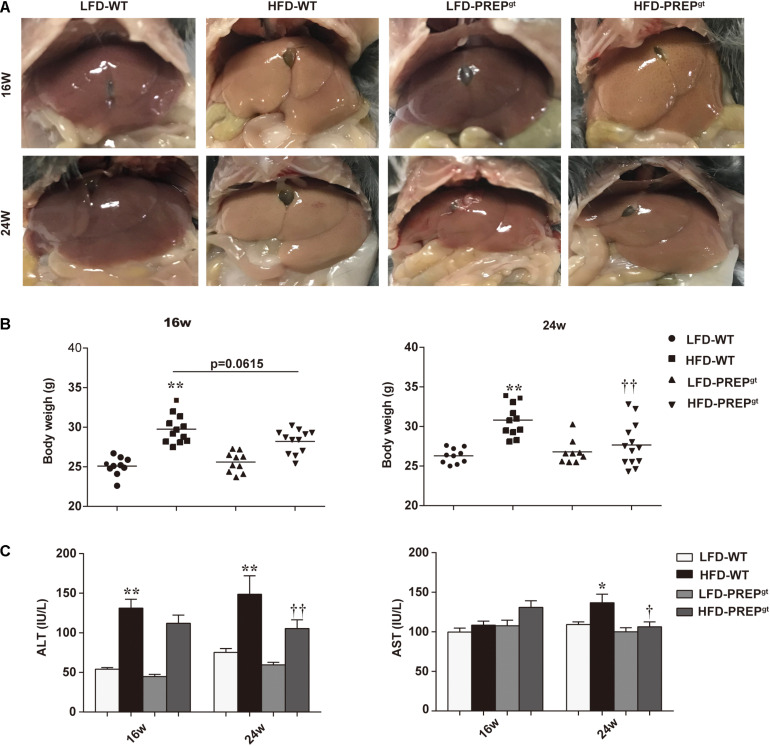
PREP disruption reduced mice weight and protected against liver injury in HFD-fed mice as NAFLD progressed. **(A)** Representative gross pictures of the livers in different groups; **(B)** Body weight was measured at 16 and 24 w, respectively; **(C)** Plasma levels of ALT and AST were measured. All data are presented as the means or mean ± SEM (*n* = 8–13). *P < 0.05 and **P < 0.01, LFD-WT vs. HFD-WT; ^†^*P* < 0.05 and ^††^*P* < 0.01, HFD-WT vs. HFD-PREP^gt^.

**FIGURE 2:**
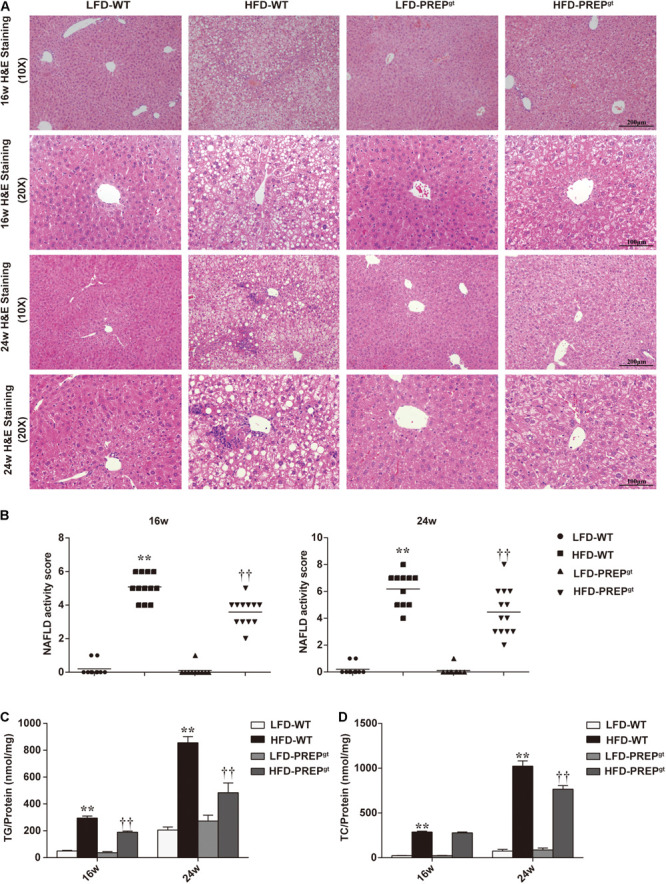
Lipid accumulation and hepatic steatosis ameliorated by PREP disruption in the liver of HFD-fed mice as NAFLD progresses. **(A)** Liver sections were harvested and stained with hematoxylin-eosin (200× magnification) at 16 and 24 w; **(B)** NAS is calculated from the semi-quantitative evaluation of hepatic steatosis, lobular inflammation, and hepatocyte ballooning in different groups; hepatic levels of triglycerides **(C)** and cholesterol **(D)** were detected in mice. All data are presented as the mean or mean ± SEM (*n* = 8–13). **P < 0.01, LFD-WT vs. HFD-WT; ^††^*P* < 0.01, HFD-WT vs. HFD-PREP^gt^.

After ingesting an HFD for 16 weeks (w), HFD-WT mice developed enlarged and yellow greasy livers compared to LFD-WT mice, while the gross picture was more evident after 24 weeks feeding (Figure 1A). The general view of the liver improved after PREP disruption (Figure 1B). The HFD-WT mice gained more body weight than LFD-WT mice, while the weights of HFD-PREP^gt^ mice decreased to varying degrees after 16 and 24 weeks feeding (5.24 and 10.26%, respectively) compared to HFD-WT mice (Figure 1B). Additionally, ALT and AST serum levels were greatly elevated in HFD-WT mice and significantly decreased in HFD-PREP^gt^ mice after 24 weeks feeding (Figure 1C). However, after 16 weeks feeding, only the ALT serum results displayed statistical significance. H&E staining demonstrated substantially increased fat accumulation in the livers of the HFD-WT mice (16 and 24 weeks) compared with that in the LFD-WT, respectively, while lobular inflammation is more evident in the 24 weeks HFD-WT mice (Figure 2A). Specifically, histological changes improved in HFD-PREP^gt^ mice at both 16 and 24 weeks, NAS decreased by 29.51 and 27.82% compared with HFD-WT mice, respectively (Figures 2A,B). Hepatic triglyceride content increased significantly at both time points with HFD feeding, while the indexes of 16 and 24 weeks HFD-PREP^gt^ mice decreased by 35.75 and 43.41% compared with HFD-WT mice, respectively (Figure 2C). Hepatic cholesterol results from both time points were similar to hepatic triglycerides to a certain extent (Figure 2D).

### PREP Disruption Dynamically Activates the AMPK/SIRT1 Pathway to Regulate Hepatic Lipid Synthesis in HFD-Induced NAFLD Mice at Different Time Points

**FIGURE 3:**
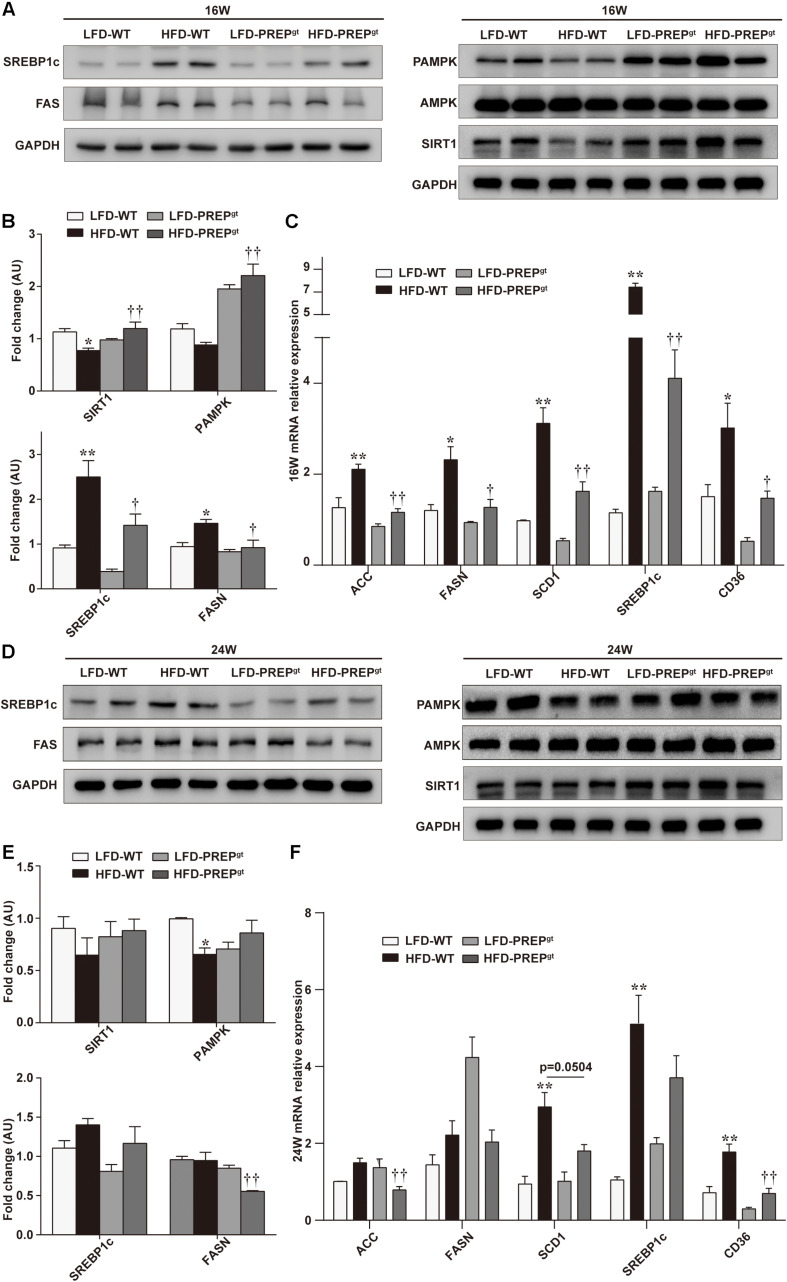
PREP disruption dynamically activates the AMPK/SIRT1 pathway to regulate hepatic lipid synthesis in HFD-fed mice as lipid accumulation progresses. **(A)** Liver expression of total and phosphorylated AMPK (PAMPK) and SIRT1 proteins and their downstream molecules (SREBP1c and FASN) were detected at 16 w in mice using western blot analysis; **(B)** relative bar graphs display blot quantification analysis for 16 w mice; **(C)** hepatic mRNA levels of lipid synthesis-associated genes of 16 w mice were examined using RT-PCR; **(D)** protein levels of PAMPK/SIRT1, SREBP1c, and FASN in livers were detected in 24 w mice; **(E)** relative bar graphs display blot quantification analysis for 16 weeks mice; **(F)** hepatic mRNA levels of lipid synthesis-associated genes of 24 w mice were examined using RT-PCR. All data are presented as the mean ± SEM (*n* = 4). *P < 0.05 and **P < 0.01, LFD-WT vs. HFD-WT; ^†^*P* < 0.05 and ^††^*P* < 0.01, HFD-WT vs. HFD-PREP^gt^.

To further understand the mechanisms that exacerbate NAFLD progression, we measured the hepatic AMPK/SIRT1 pathway’s protein levels in mice. We observed downregulation of PAMPK and SIRT1 protein expression in 16 weeks HFD-WT mice compared with LFD-WT mice at the corresponding time (25.79 and 31.63%, respectively), while significant upregulation was observed in 16 weeks HFD-PREP^gt^ mice compared with the HFD-WT mice (150.47 and 54.54%, respectively; Figures 3A,B). The differences in P62 levels and the LC3B-II/LC3B-I ratios (autophagy-related proteins) between HFD-WT mice and HFD-PREP^gt^ mice at 16 weeks display no significance (Supplementary Figure 1a). We also determined the levels of downstream factors, such as sterol regulatory element-binding protein 1c (SREBP1c) and fatty acid synthase (FASN), to evaluate PREP disruption effects on lipid metabolism. Upregulation of SREBP1c and FASN were observed in 16 weeks HFD-WT mice, while significant downregulation was observed in HFD-PREP^gt^ mice at the corresponding time compared with the HFD-WT mice (43.17 and 37.1%, respectively; Figures 3A,B). In addition, liver mRNA levels of AMPK/SIRT1-mediated lipogenesis enzymes, such as acetyl-coenzyme A carboxylase (ACC), FASN, stearoyl-CoA desaturase1 (SCD1), SREBP1c, and CD36, were lower in the 16 weeks LFD-WT mice (40.17∼84.47%) and HFD-PREP^gt^ mice (44.58∼51.23%) compared with the corresponding levels in the HFD-WT mice (Figure 3C). However, PAMPK and SIRT1 protein levels were not significantly upregulated in 24 weeks HFD-PREP^gt^ mice compared to HFD-WT mice (31.47 and 36.34%), along with the protein levels of SREBP1c and FASN, which were downregulated (16.78 and 41.46%; Figures 3D,E). Reduced P62 levels were observed in 24 weeks HFD-PREP^gt^ mice compared with HFD-WT mice, but the LC3B-II/LC3B-I ratios did not display a significant difference (Supplementary Figure 1b). However, the reduced LC3B-II/LC3B-I ratio in 24 weeks HFD-WT mice was more evident than that observed in 24 weeks HFD-PREP^gt^ mice than LFD mice, respectively (Supplementary Figure 1b). After 24 weeks, liver mRNA levels associated with the *de novo* lipogenesis pathway (ACC, FASN, SREBP1c, SCD1, and CD36) were lower in LFD-WT mice (32.38∼79.36%) and HFD-PREP^gt^ mice (7.95∼47.15%) relative to HFD-WT mice (Figure 3F). However, some differences did not display significance.

### Hepatic Inflammation and Related Signal Molecules Were Attenuated by PREP Disruption in HFD-Fed Mice

**FIGURE 4:**
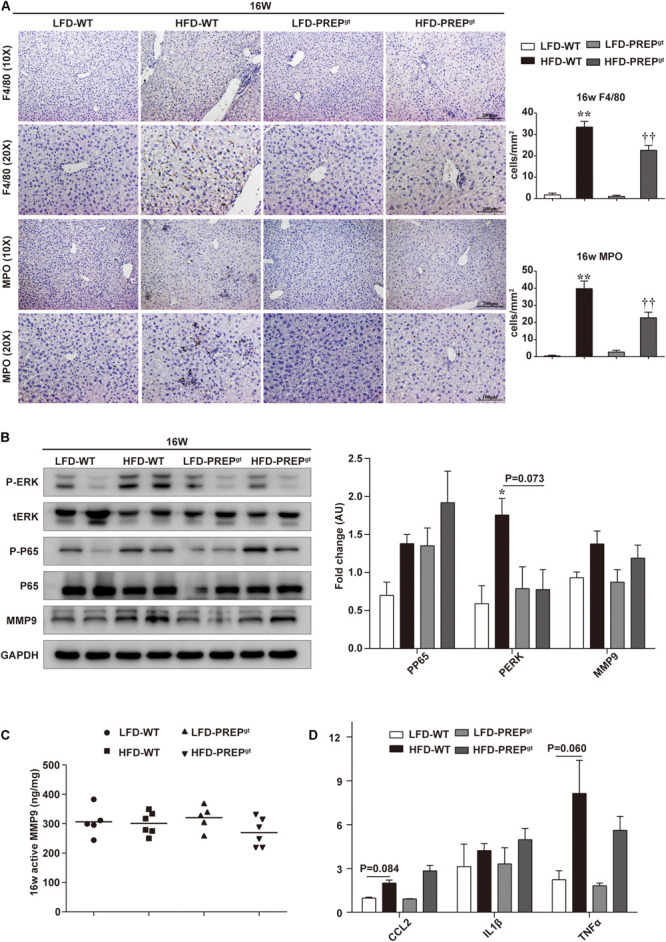
Hepatic inflammation and expression of related signaling molecules were slightly attenuated by PREP disruption in HFD-fed mice at 16 w. **(A)** Liver sections harvested from mice at 16 w were stained with anti-F4/80 and anti-MPO; **(B)** protein levels of p-ERK, p-p65, and MMP9 were detected using western blot analysis; **(C)** hepatic active MMP9 levels were measured using fluorometric assays; **(D)** mRNA expression of proinflammatory cytokines. All data are presented as the mean or mean ± SEM (*n* = 4–6). *P < 0.05 and **P < 0.01, LFD-WT vs. HFD-WT. ^††^*P* < 0.01, HFD-WT vs. HFD-PREP^gt^.

**FIGURE 5:**
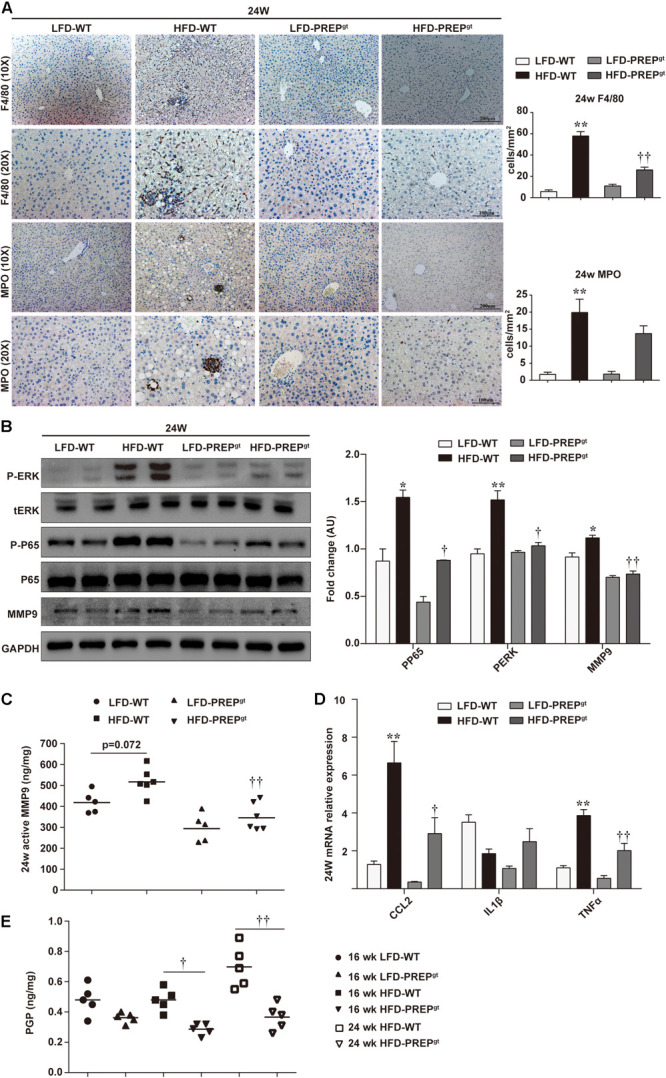
Hepatic inflammation increased as NASH progressed, while PREP disruption suppressed the detrimental microenvironment at later stages of HFD-induced NASH. **(A)** Liver sections harvested from mice after 24 w were stained with anti-F4/80 and anti-MPO; **(B)** protein levels of p-ERK, p-p65, and MMP9 were detected using western blot analysis; **(C)** hepatic active MMP9 levels were measured using fluorometric assays; **(D)** mRNA expression of proinflammatory cytokines; **(E)** Production of PGP in the liver. All data are presented as the mean or mean ± SEM (*n* = 4–6). *P < 0.05 and **P < 0.01, LFD-WT vs. HFD-WT; ^†^*P* < 0.05 and ^††^*P* < 0.01, HFD-WT vs. HFD-PREP^gt^.

Upon examination of hepatic inflammatory status, we observed that liver sections from HFD-PREP^gt^ mice contained fewer MPO-positive cells (neutrophils) and F4/80-positive cells (macrophages and Kupffer cells) compared with HFD-WT mice at 16 weeks (22.76 ± 3.31 vs. 39.76 ± 4.46, 22.62 ± 2.23 vs. 33.46 ± 13.32, respectively, Figure 4A). Moreover, the phosphorylation states of ERK (p-ERK) and nuclear factor κB (NFκB) p65 (p-p65) and related proinflammatory cytokines in livers were detected in different groups. However, differences in protein expressions of p-p65 and MMP9, including total MMP9 and its active form, were not statistically significant between HFD-WT mice and HFD-WT mice at 16 weeks (Figure 4B,C). p-ERK protein expression was significantly higher in HFD-WT mice than in LFD-WT mice, while this appeared downregulated in HFD-PREP^gt^ mice (*P* = 0.073; Figure 4B). Besides, hepatic mRNA levels of CCL2, tumor necrosis factor α (TNFα), interleukin 1β (IL1β), and IL6 were increased in the livers of HFD-WT mice and HFD-PREP^gt^ mice at 16 weeks; however, these differences were not statistically significant (Figure 4D). Interestingly, we detected the same indexes in mice’s liver at 24 weeks, while results differed in terms of hepatic inflammation progression. Liver sections from 24 weeks HFD-PREP^gt^ mice contained fewer MPO-positive cells and F4/80-positive cells compared with HFD-WT mice at 24 weeks (13.71 ± 2.28 vs. 19.88 ± 3.91, 26.05 ± 2.53 vs. 57.82 ± 4.21, respectively; Figure 5A). HFD-fed PREP^gt^ mice also showed significant decreases in the phosphorylation states of p-ERK and p-p65, and mRNA levels of CCL2 and TNFα, compared with HFD-fed WT mice at 24 weeks (Figures 5B,D). Moreover, the active form of MMP9 was downregulated in HFD-PREP^gt^ mice compared with HFD-WT mice (32.6%), consistently with its protein level (Figures 5B,C). Furthermore, PGP production of HFD-WT mice markedly increased as hepatic inflammation progressed, and it was further downregulated in the liver of 24 weeks HFD-PREP^gt^ mice compared with 24 weeks HFD-WT mice (47.56%; Figure 5E).

### PREP Gene Disruption Alleviated Gut Microbiota Dysbiosis in Mice Fed a HFD

**FIGURE 6:**
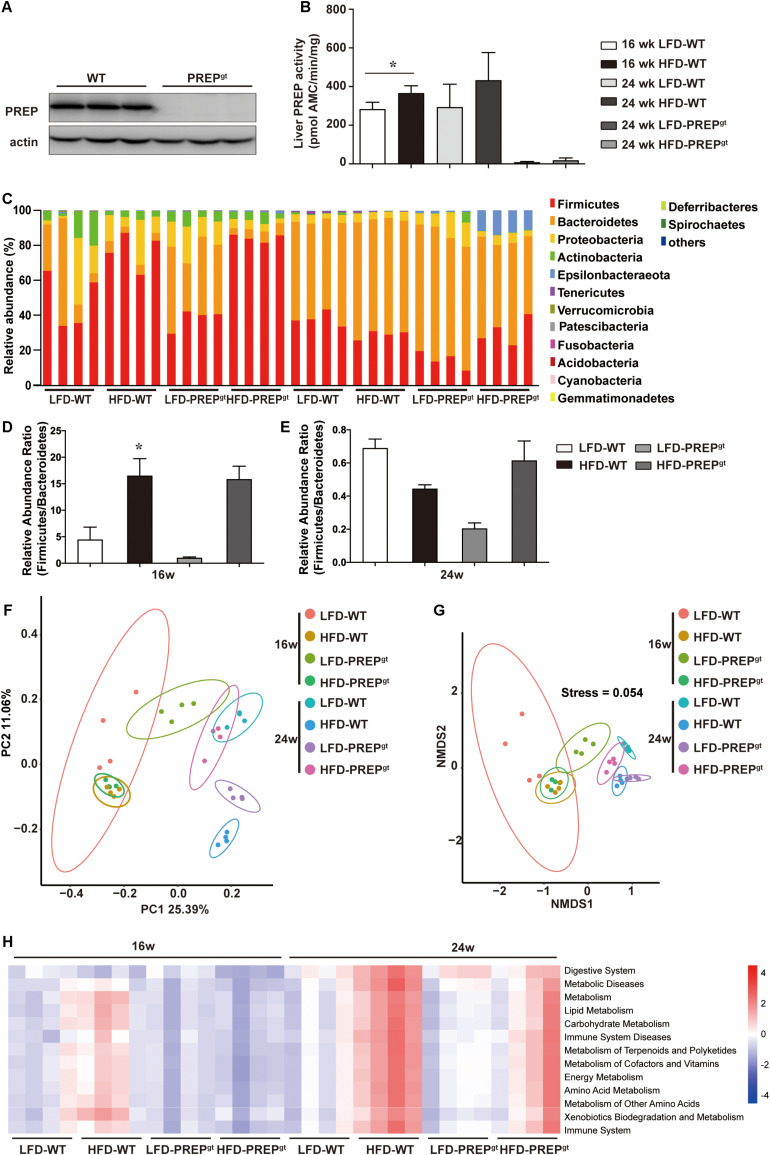
PREP gene disruption improved the gut flora structure and altered the metabolic pathways involved with the microbiota of mice fed a HFD. **(A)** Representative anti-PREP western blot images of the liver of WT and PREP^gt^ mice; **(B)** PREP enzymatic activity measured (± SEM) in liver samples from WT and PREP^gt^ mice, *P < 0.05; **(C)** relative abundance of taxa at the phylum level as 16S rRNA-based gut microbial profiling was performed; **(D)** the *Firmicutes* to *Bacteroidetes* ratio at 16 weeks mice, *P < 0.05, LFD-WT vs. HFD-WT; **(E)** the *Firmicutes* to *Bacteroidetes* ratio at 24 weeks mice, *P < 0.05, LFD-WT vs. HFD-WT; **(F)** principal coordinate analysis of an unweighted unifrac distance matrix; **(G)** non-metric multidimensional scaling analysis of an unweighted unifrac distance matrix. **(H)** prediction of the functional genes in the bacterial community in the gut, performed using PICRUSt (*n* = 4).

The PREP^gt^ mice used in this study carry a partial exon three deletion in the PREP gene, which caused complete PREP protein loss. The liver PREP activity of HFD-WT mice was higher than that of LFD-WT mice at both time points. Figures 6A,B shows representative PREP western blot images (Figure 6A) and activity measurements (Figure 6B) of the liver in mice, respectively. In PREP^gt^ mice, specific PREP activity was significantly downregulated compared with WT mice (Figure 6B).

Fecal samples were collected at 16 and 24 weeks, and the microbiota composition was analyzed using 16S rRNA gene amplicon sequencing. First, the gut microbial profile at the phylum level was assessed. At the phylum level, we observed increased *Firmicutes* abundance and decreased *Bacteroidetes* abundance in the HFD-WT mice and the HFD-PREP^gt^ mice at 16 weeks (Figures 6C,D). However, the difference in the *Firmicutes* to *Bacteroidetes* ratio between the LFD-WT, and HFD-WT mice, was not statistically significant at 24 weeks (Figures 6C,E). Non-metric multidimensional scaling analysis and principal coordinate analysis showed that the overall composition of the gut flora expectedly changed in the 24 weeks HFD-WT mice, and the microbial profile slightly shifted in 24 weeks HFD-PREP^gt^ mice (Figures 6F,G). The overall composition of the gut flora in 16 weeks HFD-WT and 16 weeks HFD-PREP^gt^ mice was similar (Figures 6F,G). However, pathways related to energy and nutrient (amino acid, lipid, and glucose) metabolism were upregulated and downregulated in HFD-WT in HFD-PREP^gt^ mice, respectively, at both time points, suggesting that PREP may affect metabolic processes by regulating the gut flora (Figure 6H).

**FIGURE 7:**
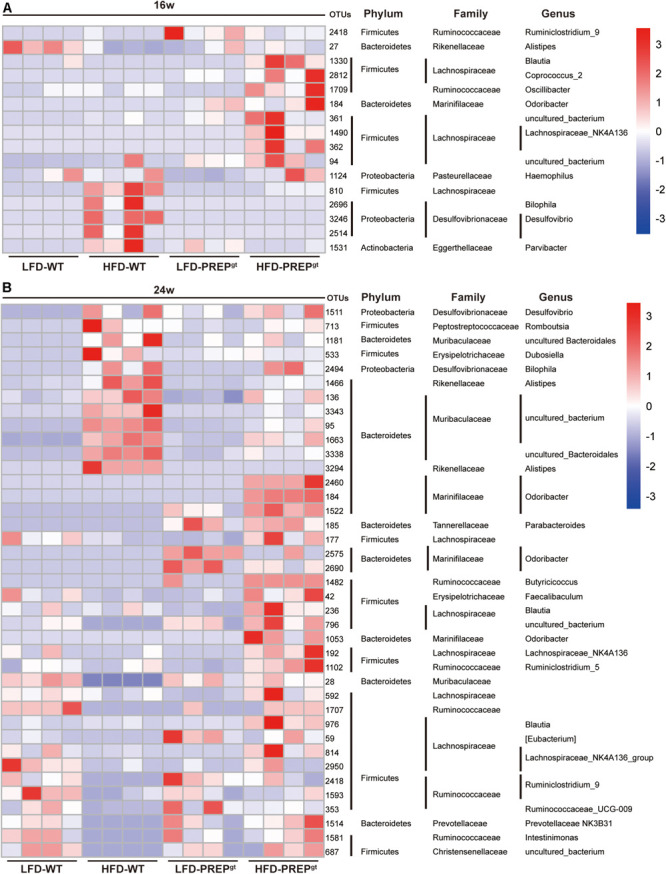
**(A)** The abundance of OTUs and representative bacterial taxa information (phylum, family, and genus) of 16 w mice (*n* = 4); **(B)** the abundance of OTUs and representative bacterial taxa information (phylum, family, and genus) of 24 w mice (*n* = 4).

After analyzing the microbial profile in detail, levels of *Ruminiclostridium 9*, *Blautia, Corprocccus 2, Lachnospiraceae NK4A139, Oscillibacter*, and *Odoribacter* increased in the 16 weeks mice (Figure 7A). The levels of *Ruminiclostridium 9*, *Blautia*, *Lachnospiraceae NK4A139, Odoribacter*, *Intestinimonas*, and *Faecalibaculum* decreased in 24 weeks HFD-WT mice and increased after PREP gene knockout (Figure 7B). *Desulfovibrio*, *Romboutsia*, and *Bilophila* increased in HFD-WT mice and decreased in HFD-PREP^gt^ mice at 16 and 24 weeks (Figures 7A,B). The expression of SCFAs receptors (GPR41 and GPR43) decreased in HFD-WT mice and slightly improved after PREP disruption at 16 and 24 weeks (Supplementary Figures 2a–d). The level of butyrate in 16 weeks HFD-PREP^gt^ mice significantly increased compared with HFD-WT mice (Supplementary Figure 2e).

### PREP Gene Disruption Diminished Damage to the Intestinal Epithelial Barrier in Mice Fed a HFD

**FIGURE 8:**
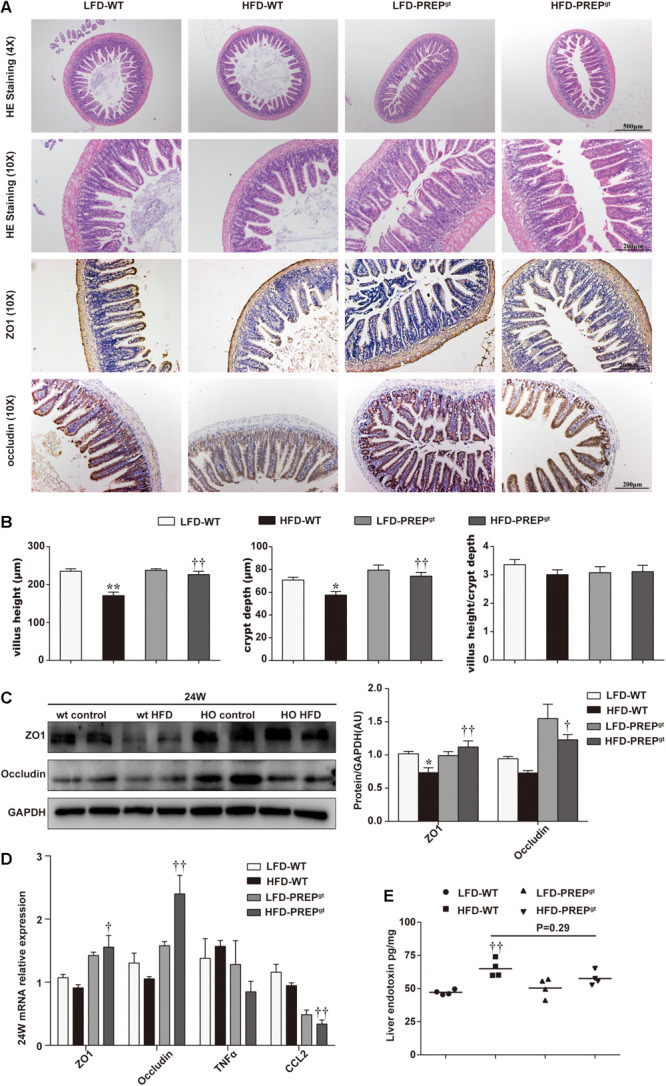
Damage to the intestinal epithelial barrier in mice fed a HFD was reversed by PREP gene knockout. **(A)** Representative H&E staining and immunostaining of ZO-1 and occluding of the terminal ileum sections are shown; **(B)** as the villus height and the depth of the crypts were measured, the ratio of villus height to crypt depth was calculated; **(C)** protein levels of ZO-1 and occludin in the intestine were measured using western blot. Bar graphs display the quantification of western blots; **(D)** intestinal mRNA levels of tight junction protein-associated genes and inflammatory factors were examined using RT-PCR; **(E)** the hepatic level of endotoxin was measured using a mouse lipopolysaccharide ELISA kit. All data are presented as the mean or mean ± SEM (*n* = 4–6). *P < 0.05 and **P < 0.01, LFD-WT vs. HFD-WT; ^†^*P* < 0.05 and ^††^*P* < 0.01, HFD-WT vs. HFD-PREP^gt^.

We explored whether PREP loss exerted beneficial effects on the integrity of the intestinal barrier in mice under HFD stimulation. As shown in Figure 8A, we observed abnormal morphological alterations of intestinal mucosa in HFD-WT mice compared with LFD-WT mice and HFD-PREP^gt^ mice. A loss of normal villus structure in the terminal ileac epithelium was observed in HFD-WT mice (Figure 8A). The villus height and crypt depth were significantly decreased in the terminal ileum of HFD-WT mice compared with LFD-WT mice and HFD-PREP^gt^ mice; however, changes in the villus to crypt ratio were not evident (Figures 8A,B). Besides, we detected protein and mRNA expression levels of zonula occludens 1 (ZO1) and occludin. Protein levels of ZO1 and occludin were reduced in the intestine of HFD-WT mice compared with LFD-WT mice, and the two were increased in HFD-PREP^gt^, as shown by immunostaining and immunoblots (Figures 8A,C). mRNA expression of ZO1 and occludin were consistent with protein expression, respectively (Figure 8D). As shown in Figure 8E, elevated liver endotoxin levels were significantly increased in HFD-WT mice compared with LFD-WT mice. Although the indexes were slightly decreased in HFD-PREP^gt^ mice, this difference was not statistically significant (Figure 8E).

## Discussion

The involvement of the gut-liver axis in the pathogenesis and progression of NAFLD is increasingly being recognized. PREP action is quite complex, as it can induce metabolic benefits or proinflammatory damage to the intestinal environment (Koelink et al., 2014; Olivares et al., 2019). Our previous study found that PREP disruption plays a beneficial role in NAFLD progression, mainly through decreases in the number of chemokines (such as PGP) and inflammatory cell accumulation (Jiang et al., 2020). Notably, different hepatic pathogenesis mechanisms have previously been described, and complex PREP actions have been reported; therefore, we carried out this study to imitate the different NAFLD stages to uncover PREP’s influence on the mice gut. We found that (Younossi et al., 2016) hepatic lipid metabolism improved dynamically by activating the SIRT1/AMPK pathway via PREP disruption under HFD feeding conditions; (Saltzman et al., 2018) PREP disruption markedly improved hepatic proinflammatory status progression by inhibiting phosphorylated ERK and p65 as NASH progresses to more severe stages; (Aron-Wisnewsky et al., 2020) PREP disruption improves intestinal dysbiosis and protects against intestinal epithelial barrier damage induced by a HFD. Therefore, we provided compelling evidence to demonstrate that PREP inhibition effects may play various roles in NAFLD progression.

Numerous studies revealed that regulating the gut dysbiosis contributed to restraining NAFLD development (Si et al., 2018; Wang X. et al., 2019; Aron-Wisnewsky et al., 2020). When the gut microbiota is in dysbiosis, the host’s health is compromised as the gut microbiota is unable to maintain control of local homeostasis, thereby increasing intestinal permeability (Saltzman et al., 2018). The modulation of the gut microbial profile by PREP disruption may prevent NAFLD by altering the relative abundance of several “beneficial indicators” in the cecum, thereby promoting homeostasis. *Odoribacter* and *Oscillibacter* are closely associated with intestinal epithelial homeostasis, while *Lachnospiraceae NK4A139* negatively correlated with serum lipid levels (Zhao et al., 2019; Mu et al., 2020). Besides, *Ruminiclostridium 9* belongs to the *Ruminococcaceae* family; reportedly, a high abundance of *Ruminococcaceae* in the cecum could effectively prevent malnutrition (Million et al., 2016). The short-chain fatty acid butyrate-producing bacteria, such as *Ruminococcaceae*, *Odoribacter*, *Intestinimonas*, and *Faecalibaculum* (Kang et al., 2021; Liu et al., 2021). Notably, the abovementioned bacteria were more abundant in HFD-PREP^gt^ than HFD-WT mice.

PREP inhibition may benefit flora homeostasis by suppressing protein fermentation, which reduces indole and phenol production, thus preventing the thinning of the intestinal mucous barrier. In addition, increased phosphorylation and activation of AMPK and its downstream lipid metabolism targets (Chiu et al., 2015) in the liver are associated with butyrate-producing bacteria (Leung et al., 2016). Butyrate bounds to endogenous GPR41- and GPR43-containing receptors in the liver, impacting lipid *de novo* synthesis (Lu et al., 2016). We only detected butyrate levels in the colon of 16 weeks mice; however, a previous study reported that sodium butyrate could delay the onset of early signs of NAFLD in mice (Jin et al., 2016). Our previous studies also indicated that PREP is closely related to energy metabolism and the downstream lipid metabolism targets of AMPK (Jiang et al., 2020). The functional consequences of this taxa shift and our previous work provide clues about how PREP inhibition may regulate flora homeostasis, trigger the AMPK signaling pathway, and improve liver lipid metabolism. Protective intestinal microbiota also associates other metabolites such as the specific bile acids, which promotes protection against NAFLD (Petrov et al., 2019). However, the potential relationship between the bile acids and PREP warrants further study.

Our study detected phosphorylated AMPK (PAMPK) in the liver after 24 weeks HFD feeding. The changes were not evident as the activation of PAMPK appeared slightly elevated with no statistical significance alongside its downstream molecules (SIRT1/SREBP1c/FASN (Teng et al., 2019)), although we observed a noticeable improvement in lipid accumulation in PREP knockout mice after 24 weeks HFD feeding. It is known that lipogenesis can be promoted by SIRT1-mediated inhibition of AMPK phosphorylation and activation, leading to hepatic steatosis (Srivastava et al., 2012; Teng et al., 2019). We should further consider that PREP expression levels, protein distribution, and activity correlate with aging and are reported in many neurodegenerative conditions (Svarcbahs et al., 2019). Besides, aging promotes the development of diet-induced murine steatohepatitis, but not steatosis (Fontana et al., 2013), and hepatic steatosis and inflammation may contribute to the development of NAFLD via different pathways, respectively (Mahli et al., 2018). We hypothesized that PREP might affect NAFLD progression at different time points. To verify our conjecture, we investigated the early stage of NASH – before the 24-week HFD feeding model. Interestingly, activation of PAMPK/SIRT1 and improvements in lipid metabolism were pronounced in the liver of 16 weeks HFD-PREP^gt^ mice. Autophagy could be activated through PREP inhibition via protein phosphatase 2A in the brain (Svarcbahs et al., 2020). Further, autophagy may be mediated directly by the AMPK/SIRT1 pathway in hepatic steatosis (Wang Y. et al., 2019). As NASH progressed, we observed apparent autophagy damage in 24 weeks HFD-WT mice compared with LFD-WT mice; however, this improved upon PREP gene knockout. However, it is unclear which pathway is responsible for the dynamic autophagy changes observed during NASH, and further research on the subject is warranted. Since PREP disruption improved intestinal flora disorders and results showed the liver in different NAFLD stages, we concluded that PREP inhibition might improve lipid metabolism via the PAMPK/SIRT1 pathway in early NAFLD stages when lipogenesis plays a major role compared to inflammation.

However, hepatic lipotoxicity and inflammation are not easily separated, as hepatic lipotoxicity-induced wound healing requires subsequent inflammation, remodeling of the hepatic vasculature and matrix, and outgrowth of liver progenitors (Suzuki and Diehl, 2017). Tissue outside the liver (such as adipose tissue or the gut) and processes within the organ (for instance, lipotoxicity) contribute to NASH development (Schuster et al., 2018). Besides tracking the lipid metabolic benefits from gut dysbiosis improvement, our results indicated that PREP gene disruption attenuates mucosal lesions caused by HFD feeding. Dysbiosis increases gut permeability to bacterial products and increases hepatic exposure to injurious substances that increase hepatic inflammation and fibrosis (Leung et al., 2016). Notably, *Bilophila* and *Desulfovibrio* are gram-negative endotoxin-producing bacteria known to increase intestinal permeability and circulate gut-derived antigens, primarily LPS (Moreno-Indias et al., 2016; Zhuang et al., 2020).

**FIGURE 9:**
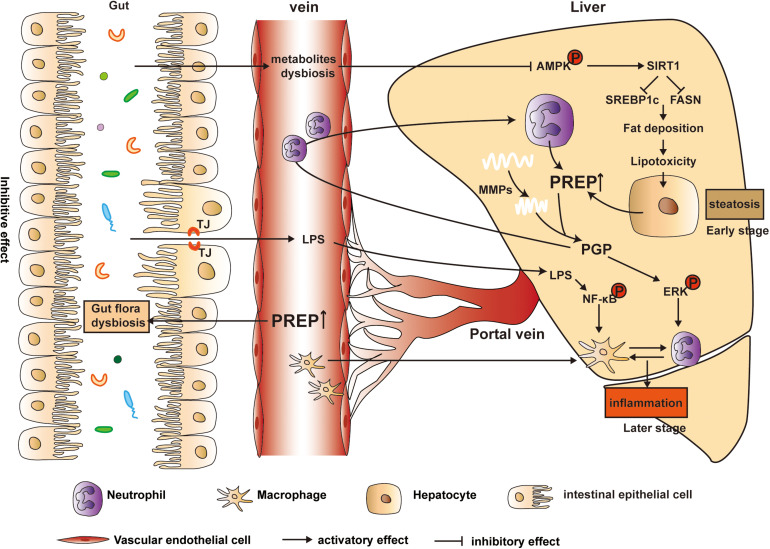
Model of the proposed mechanism underlying PREP disruption-mediated dynamic amelioration of hepatic steatosis and inflammation via intestinal dysbiosis regulation, AMPK/SIRT1 pathway activation, and inflammatory signaling pathway inhibition.

On the one hand, compared with the 24 weeks HFD-WT mice, the corresponding HFD-PREP^gt^ mice display a lower abundance of *Desulfovibrio* and decreased hepatic LPS content, although this was not statistically significant. On the other hand, a former study indicated that PREP might play a role in microglial activation since PREP knockout mice lack a response to LPS (Hofling et al., 2016). Therefore, we hypothesize that the combined effects of PREP disruption on gut dysbiosis and its response to inflammatory triggers inhibit NASH progression. Interestingly, factors associated with proinflammation and its related signaling molecules showed dynamic changes in HFD-PREP^gt^ mice upon hepatic inflammation progression. Our previous works demonstrated that PREP could potentially affect the progression of hepatic inflammation, possibly by regulating chemotactic factors (such as PGP and MMPs) (Jiang et al., 2020), and in this study, we found this effect was more important and evident in the later and more severe stage of NAFLD. This observation may be partially explained by the fact that NASH is considered a potentially progressive disorder, as liver inflammation may prompt collagen matrix synthesis and deposition (Suzuki and Diehl, 2017), which PREP and MMP9 hydrolyze to produce PGP (chemotaxis of neutrophils) (Weathington et al., 2006; Gaggar et al., 2008). Nevertheless, endotoxin-mediated TLR4/NF-κB pathway activation in macrophages reportedly plays a pivotal role in NASH pathogenesis (Zhao et al., 2019). PREP increased PGP production, possibly activating ERK and facilitating crosstalk between neutrophils, which release MPO and lipocalin2, and macrophages to exacerbate their migration and activation (Ye et al., 2016; Jiang et al., 2020). Based on the above, our findings demonstrate that HFD-induced NAFLD status in mice was alleviated to varying degrees by PREP disruption, contributing to the remission of gut flora dysbiosis and hepatic inflammation (Figure 9).

Our study also has some limitations. First, the specific PREP deletion mechanisms (such as in the liver and gut) in mice need to be further explored. We believe that their complexity and heavy workload warrant further study. Second, we have reported the effect of PREP-specific inhibitors (S17092) on lipid synthesis *in vitro* (Zhou et al., 2016). A PREP inhibitor suitable for use in vivo experiments is still in progress. Third, we did not explore the complex relationship between PREP, autophagy, and the microbiota during NAFLD progression, which warrants future studies.

## Conclusion

In summary, PREP disruption may target multiple detrimental hepatic mechanisms related to systems, including the liver, macrophages, neutrophils, the gut, and microbiota, which may show dynamic changes during NAFLD progression. Our study demonstrates that PREP disruption dynamically ameliorates hepatic steatosis and inflammation by regulating intestinal dysbiosis, activating the AMPK/SIRT1 pathway, and inhibiting the inflammatory signaling pathway. Therefore, targeting PREP may be a viable therapeutic or preventive approach for the management of NAFLD.

## Data Availability Statement

The raw data supporting the conclusions of this article will be made available by the authors, without undue reservation.

## Ethics Statement

The animal study was reviewed and approved by all animal experiments followed the National Research Council’s Guide for the Care and Use of Laboratory Animals and were approved by the Institutional Animal Care and Use Committee of SHRM (SHRM-IACUC-022).

## Author Contributions

DJ and JZ performed most experiments. DJ and SL analyzed and interpreted the data. YC, YW, and JF designed and coordinated the research. DJ and YC drafted the manuscript. All authors contributed to the article and approved the submitted version.

## Conflict of Interest

The authors declare that the research was conducted in the absence of any commercial or financial relationships that could be construed as a potential conflict of interest.
