# Supplementary figures and images for: Prolyl Endopeptidase Gene Disruption Improves Gut Dysbiosis and Non-alcoholic Fatty Liver Disease in Mice Induced by a High-Fat Diet
Source: Front Cell Dev Biol. 2021 May 20;9:628143. doi: 10.3389/fcell.2021.628143 (PMC8172602; doi:10.3389/fcell.2021.628143)

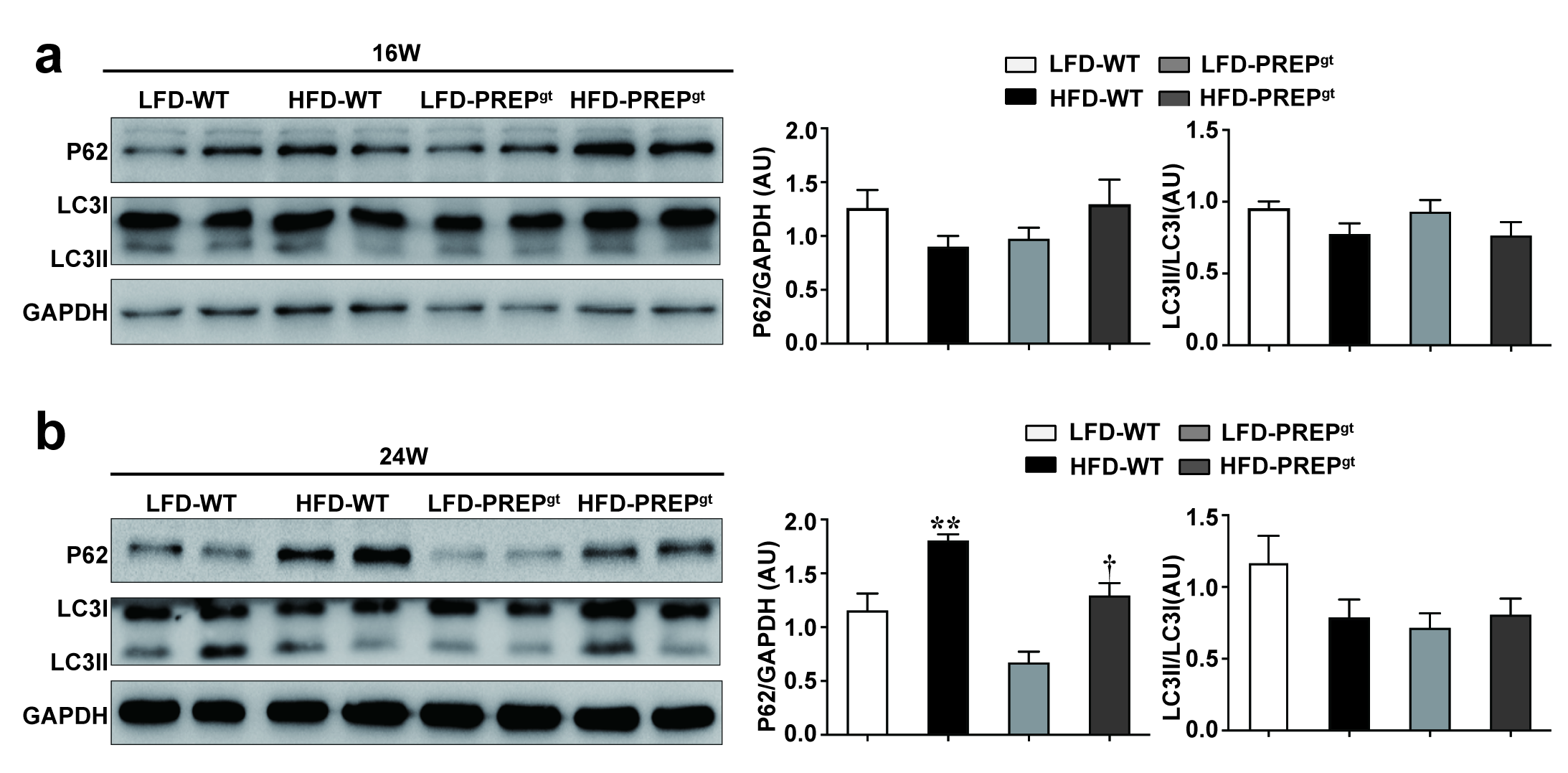

Supplement: Supplementary file 1 [file Image_1.TIF]

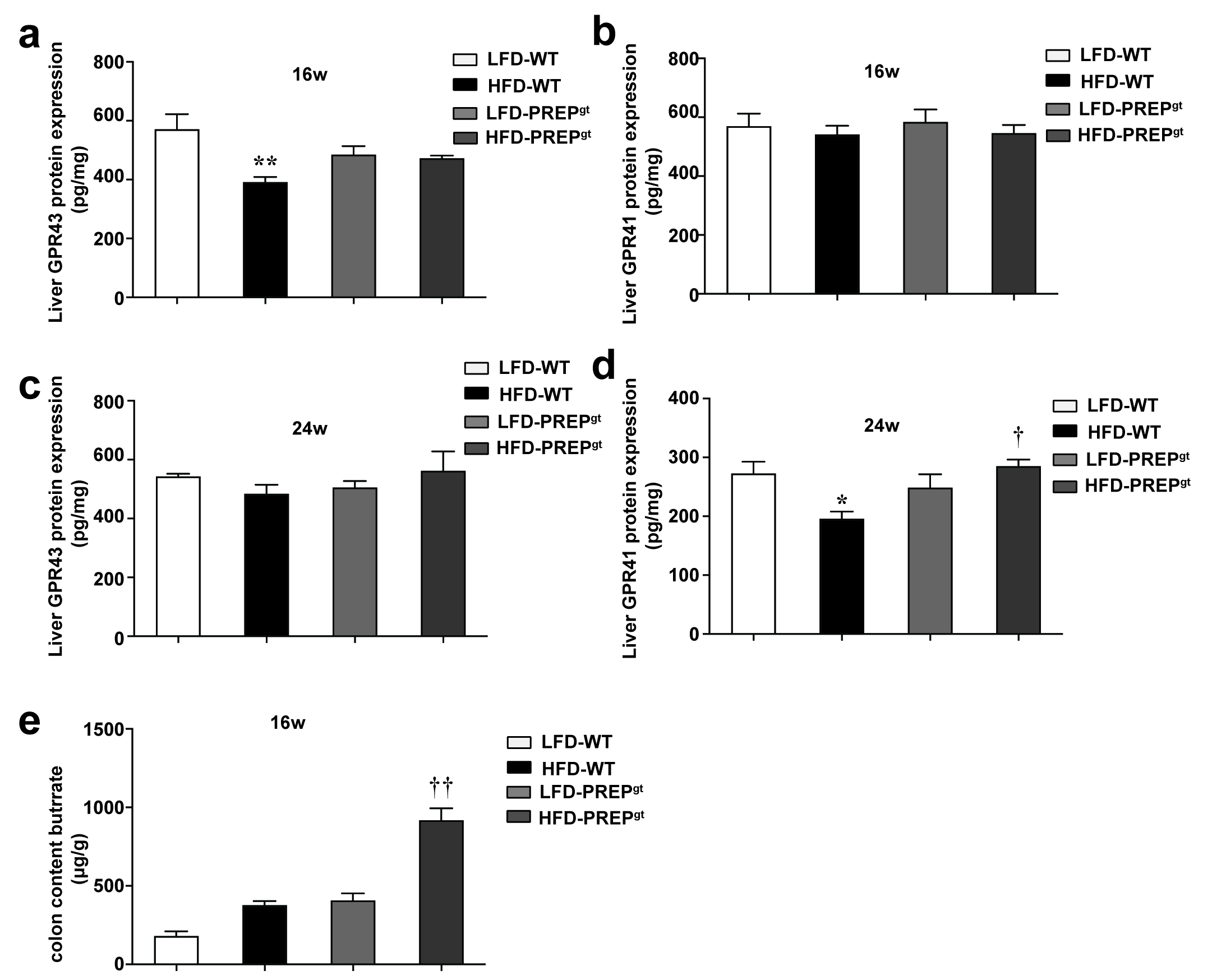

Supplement: Supplementary file 2 [file Image_2.TIF]

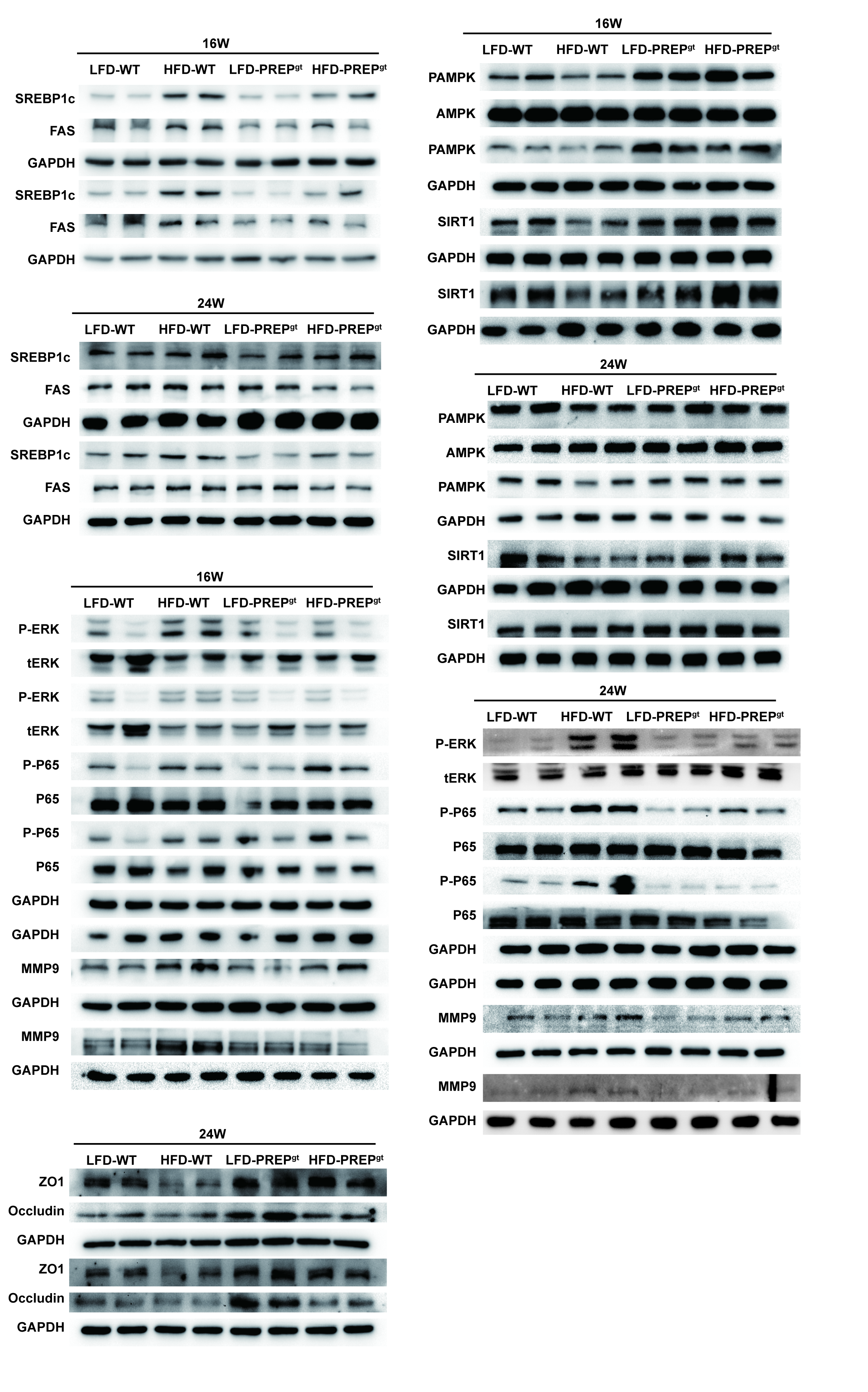

Supplement: Supplementary file 3 [file Image_3.TIF]

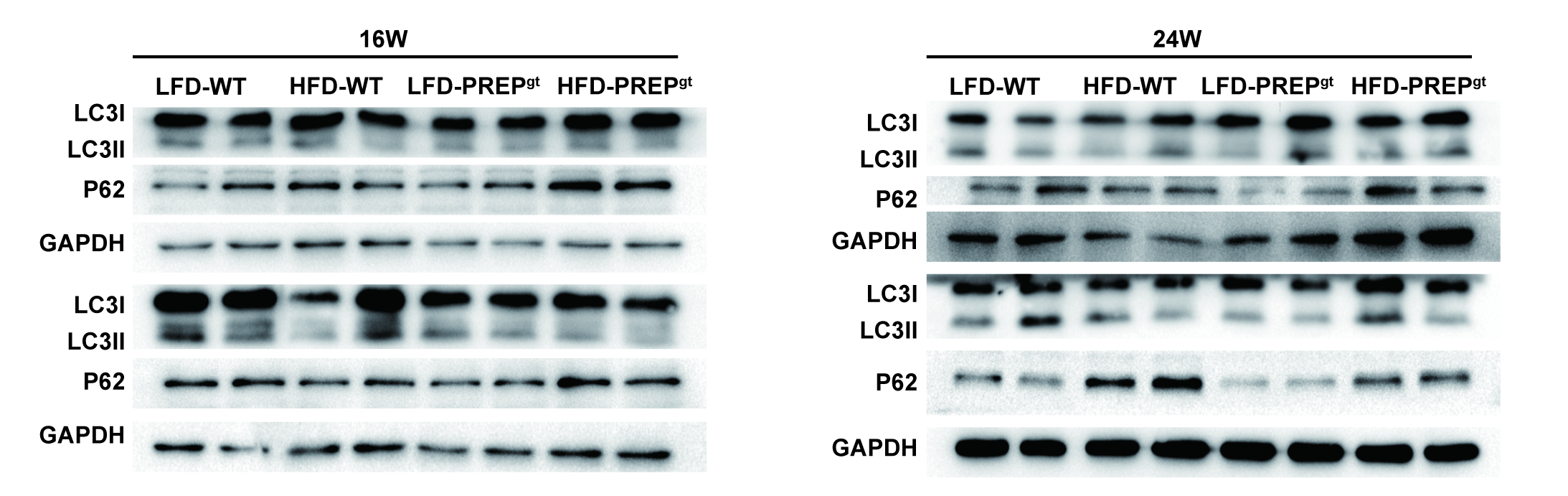

Supplement: Supplementary file 4 [file Image_4.TIF]
