# Supplementary material for: Prolyl Endopeptidase Gene Disruption Improves Gut Dysbiosis and Non-alcoholic Fatty Liver Disease in Mice Induced by a High-Fat Diet
Source: Front Cell Dev Biol. 2021 May 20;9:628143. doi: 10.3389/fcell.2021.628143 (PMC8172602; doi:10.3389/fcell.2021.628143)
Supplement: Supplementary file 5 [file Table_1.DOCX]

Table S1.List of Primer Sequences Used for RT-PCR

| Gene | Forward primer | Reverse primer |
| --- | --- | --- |
| ACC | 5′-TTGAAGGCACAGTGAAGGCTTACG-3′ | 5′-CCATCTTCCTCTGTCAGTTGCTTCTC-3′ |
| SREBP1c | 5′-GCGGCGGTTGGCACAGAG-3′ | 5′-CCTCCTCCTCAGACTGCGATCC-3′ |
| SCD1 | 5′-CTACACCTGCCTCTTCGGGA-3′ | 5′-CACGTCATTCTGGAACGCCA-3′ |
| FASN | 5′-ACCACCCAGAAGTCCCAACA-3′ | 5′-CCCTGGAACTGAGGGCCATA-3′ |
| CD36 | 5′-GCGACATGATTAATGGCACAGACG-3′ | 5′-CCGAACACAGCGTAGATAGACCTG-3′ |
| CCL2 | 5′-CCACTCACCTGCTGCTACTCATTC-3′ | 5′-CTGCTGCTGGTGATCCTCTTGTAG-3′ |
| TNF-α | 5′-GCGACGTGGAACTGGCAGAAG-3′ | 5′-GAATGAGAAGAGGCTGAGACATAGGC-3′ |
| IL-1β | 5′-TTCAGGCAGGCAGTATCACTCATTG-3′ | 5′-ACACCAGCAGGTTATCATCATCATCC-3′ |
| ZO1 | 5′-CTGGTGAAGTCTCGGAAAAATG-3′ | 5′-CATCTCTTGCTGCCAAACTATC-3′ |
| Occludin | 5′-TGCTTCATCGCTTCCTTAGTAA-3′ | 5′-GGGTTCACTCCCATTATGTACA-3′ |
